# Digital surgery group versus traditional experience group in head and neck reconstruction: a retrospective controlled study to analyze clinical value and time-economic-social effect

**DOI:** 10.1186/s12957-022-02677-0

**Published:** 2022-06-30

**Authors:** Ronghao Sun, Yuqiu Zhou, Michelle Z. Malouta, Yongcong Cai, Chunyan Shui, Li Zhu, Xu Wang, Jingqiang Zhu, Chao Li

**Affiliations:** 1 Department of Thyroid & Parathyroid Surgery, West China Hospital, Sichuan University, Chengdu, 610041 China; 2 Department of Head and Neck Surgery, Sichuan Cancer Hospital and Institute, Sichuan Cancer Center, School of Medicine, University of Electronic Science and Technology of China, Chengdu, 610041 China; 3 Department of Psychiatry, Bloomington Meadows Hospital, 3600 N Prow Rd, Bloomington, IN 47404 USA; 4 Department of Head and Neck Surgery, Chengdu Renpin Otolaryngology Hospital, Chengdu, 610000 China

**Keywords:** Head and neck tumors, Digital surgical technique, Reconstruction

## Abstract

**Objective:**

Discuss the application value of digital surgical technology in the reconstruction of head and neck defects after tumor resection and comprehensively evaluate time-economic-benefit cost.

**Methods:**

A retrospective analysis of head and neck cancer patients who underwent reconstructive operations in head and neck surgery at Sichuan Cancer Hospital from January 2015 to January 2021 was performed. According to the inclusion and exclusion criteria, a total of 52 cases were included, including 25 cases using digital surgery (DS) and 27 cases using the conventional surgery (CS). The clinical-pathological characteristics, postoperative complications, functional aesthetic evaluation indexes, and time-cost-satisfaction evaluation indexes between the two groups were compared and statistically analyzed. Typical cases using digital surgery were shared.

**Results:**

Outcomes between the two groups were comparable, and there was no significant difference in survival outcome and follow-up time between the two groups (*P* > 0.05). There was no significant difference between the two groups in the defect size, pathological type, other major clinicopathological features, or operation-related indicators (*P* > 0.05). The incidence of titanium plate displacement, deformation or exposure, and facial scar deformity in the DS group was significantly lower than that in the CS group (*P* < 0.05). However, there was no significant difference in other short-term or long-term complications (*P* > 0.05). The incidence of dysphagia and eating disorders in the DS group was significantly reduced (*P* < 0.05). The speech and social functions were improved, but not significantly (*P* > 0.05). Meanwhile, there was no significant difference in the evaluation index of facial aesthetics in this study (*P* > 0.05). Furthermore, the total operation time, preparation time of bone flap from the donor site, osteotomy time, and reconstruction time in the DS group were significantly lower than those in the traditional operation group (*P* < 0.05), but the shaping time and vascular anastomosis time of recipient area could not be shortened (*P* > 0.05). In addition, there was no significant difference in total hospitalization days between the DS group and CS group (*P* > 0.05), but the time of ICU treatment and postoperative intravenous nutrition support in the DS group were shorter than those in the CS group (*P* < 0.05). In particular, the preoperative doctor-patient communication of the DS group was more effective, and the treatment satisfaction of patients including their families was higher after operation (*P* < 0.05).

**Conclusion:**

Comprehensive application of digital surgical technology (CAD, CAM, VR, MA, etc.) in the reconstruction of the head and neck after tumor resection is feasible in clinical practice, which can not only improve the accuracy of repair, decrease some surgical complications, better preserve and improve patient’s diet and speech function, and reduce the operation and hospitalization time, but also increase the treatment cost. Furthermore, it is conducive to doctor-patient communication and improves patient satisfaction.

## Introduction

The anatomical position of the head and neck is unique, with complex functional areas, especially the oral cavity, as an important organ of speech, swallowing, and facial contours of the human body, which occupies a pivotal position. The purpose of reconstruction after head and neck tumor resection is not only to restore the anatomy but also more important to retain its proper physiological function. Especially when the tumor infiltrates hard tissue such as the maxilla and mandible, the defect after resection not only affects the appearance of patients, but also causes physiological dysfunction in chewing, swallowing, and speech, which seriously affects the quality of life and social activities of patients, resulting in heavy psychological burden and mental pressure. With the concept of personalized and functional reconstruction, people put forward higher requirements for the functional recovery of postoperative appearance and occlusion [1]. The traditional repair method is mainly made by surgeons according to the imaging data and clinical examination results. During the operation, autogenous bone transplantation and vascularized free flap repair are carried out according to the experience of the surgeons. This treatment method is an “experience dependent” treatment process, which lacks personalized design and accurate surgical guidance. The treatment process depends on the surgeon’s experience and skill level. Therefore, it is difficult to repair these defects accurately at the anatomical level and restore their original physiological functions.

Digital surgery (DS) includes three-dimensional reconstruction, computer-aided design (CAD), computer-aided manufacturing (CAM), 3D printing technology, image-guided surgical navigation, virtual reality (VR), augmented reality (AR), and mixed reality (MR) and other advanced digital technologies integrated with traditional surgery. This is a new technology based on the continuous development and progress of information technology [2–4]. Its advantages include providing a three-dimensional physical model for clinicians, visual surgical design and simulation before operation, assisting in the production of complex surgical schemes, and quantitative evaluation of surgical effect after surgery. Through intraoperative navigation technology, the operation can be carried out accurately according to the preoperative design. In recent years, with its rapid development, many studies [5–7] have proved that the application of the above technology has an important auxiliary role in the repair and reconstruction of head and neck tumors after surgical resection. It can assist in the design and operation of head and neck surgery, improve the quality of 3D reconstruction, improve the accuracy of navigation or guide plate-assisted surgery, control the negative surgical margin, and reduce iatrogenic injury. It can improve the accuracy of maxillofacial repair, enhance the accuracy of operation, save operation time, and improve the curative effect. This gives full play to the characteristics of evidence-based, quantitative, visualization and controllable of precision surgery.

In recent years, our department mainly used three-dimensional reconstruction, CAD/CAM technology, VR, and MR to assist the whole process of surgical treatment of head and neck tumors involving maxillary and mandibular reconstruction and achieved significant clinical results [8]. The aim of this study was to comprehensively evaluate the effectiveness, accuracy, and economy of these digital techniques in assisting head and neck reconstruction.

## Materials and methods

### Clinical materials

A retrospective analysis was conducted on the patients who underwent head and neck tumor resection and reconstruction in the department of head and neck surgery of Sichuan Cancer Center from January 2015 to June 2021. According to the inclusion and exclusion criteria, 52 patients with complete data were included in the study. Based on whether digital surgical techniques were used or not, they were further divided into the digital surgery group (DS group) and the conventional surgery group (CS). Among them, 25 patients in the DS group chose applicable digital surgical technology to assist the implementation of repairment and reconstruction according to the nature, scope, and location of the lesions. Three-dimensional visualization technology was used in all patients of this group, to evaluate tumor and operation risk and to stimulate the operation plan. At the same time, 3D printing technology was used to make equal scale models for preoperative evaluation, doctor-patient communication, pre-bending titanium, and osteotomy plate. Then, precise osteotomy, titanium plate fixation, and molding were carried out during the operation. For some cases with tumor involving important blood vessels, nerves, and tissue structure, VR was used to evaluate the invasion range and safe boundary of operation before operation. During the operation, MR technology was used to accurately locate the tumor position and judge the adjacent anatomical relationship of the tumor, the distance between the surgical instruments and the tumor, and the scope of surgical resection. In the CS group, 27 patients underwent empirical reconstruction based on preoperative two-dimensional imaging evaluation and intraoperative subjective judgment. The main epidemiological information and clinicopathological features of the two groups are shown in Table 1.

**Table 1:** Baseline clinicopathological characteristics of the comparison of parameters between two groups of patients

Variable	Digital surgery-assisted (***n*** = 25)	Conventional surgery (***n*** = 27)	***P***-value
**Mean age (years)**	45.7	48.8	0.52
**Gender**			0.10
Male	11	18	
Female	14	9	
**Pathological type**			0.09
Squamous cell carcinoma	7	12	
Sarcoma	3	8	
Ameloblastoma	7	3	
Others	8	4	
**Lesion location**			0.29
Maxilla	3	8	
Mandible	19	16	
Maxillofacial soft tissue	3	3	
**Flap selection**			0.19
Free fibula	12	8	
Free ilium	9	10	
Floating rib	1	0	
Composite reconstruction	3	9	
**Coexisting other conditions or diseases**			0.51
Yes	17	16	
No	8	11	
**Neck dissection**	17	15	0.35
**Re-exploration**	7	9	0.68
**Adjuvant therapy**			0.27
Yes	9	6	
No	16	21	
**Smokers**	10	15	0.26
**Number of osteotomies (mean)**	2.16	2.03	0.54
≤1	4	7	0.67
2	13	12	
≥3	8	8	
**Defect range (cm, mean)**	6.20	5.74	0.36
**Follow-up (months, mean)**	32.8	39.1	0.42
**Survival outcome**			0.12
Living	22	19	
Deceased	3	8	

### Inclusion and exclusion criteria

Inclusion criteria are as follows: (1) All cases are patients with head and neck tumors requiring surgery. (2) Pathological types are squamous cell carcinoma, sarcoma, ameloblastoma, and other diseases requiring surgical resection. (3) Four groups of surgeons with similar clinical working years and similar surgical techniques. (4) Free fibula, iliac bone, rib, or other free flaps were used for head and neck reconstruction in stage I after primary tumor resection. (5) Microsurgical vascular anastomosis was performed at least twice and a vascular stapler was used at most one time. (6) CAD, 3D printing, VR, AR, or MR techniques were performed in the DS group. (7) The data of included cases were collected and sorted for analysis. (8) All cases were followed up for at least 6 months. (9) Patients may have other basic complications (such as diabetes, high blood pressure, etc.) that do not affect the operation process. (10) There was no other adjuvant therapy except less than 2 times of neoadjuvant chemotherapy before operation.

Exclusion criteria are as follows: (1) Patients with unresectable head and neck tumors. (2) The operation process did not involve the reconstruction of a free bone flap or skin flap. (3) Incomplete data collection and collation of the included cases. (4) Age less than 18 years old or more than 70 years old. (5) Follow-up time less than 6 months or loss of follow-up. (6) Presence of other severe basic diseases affecting the operation or prognosis of patients.

### The application process of digital surgical technology

**Fig. 1:**
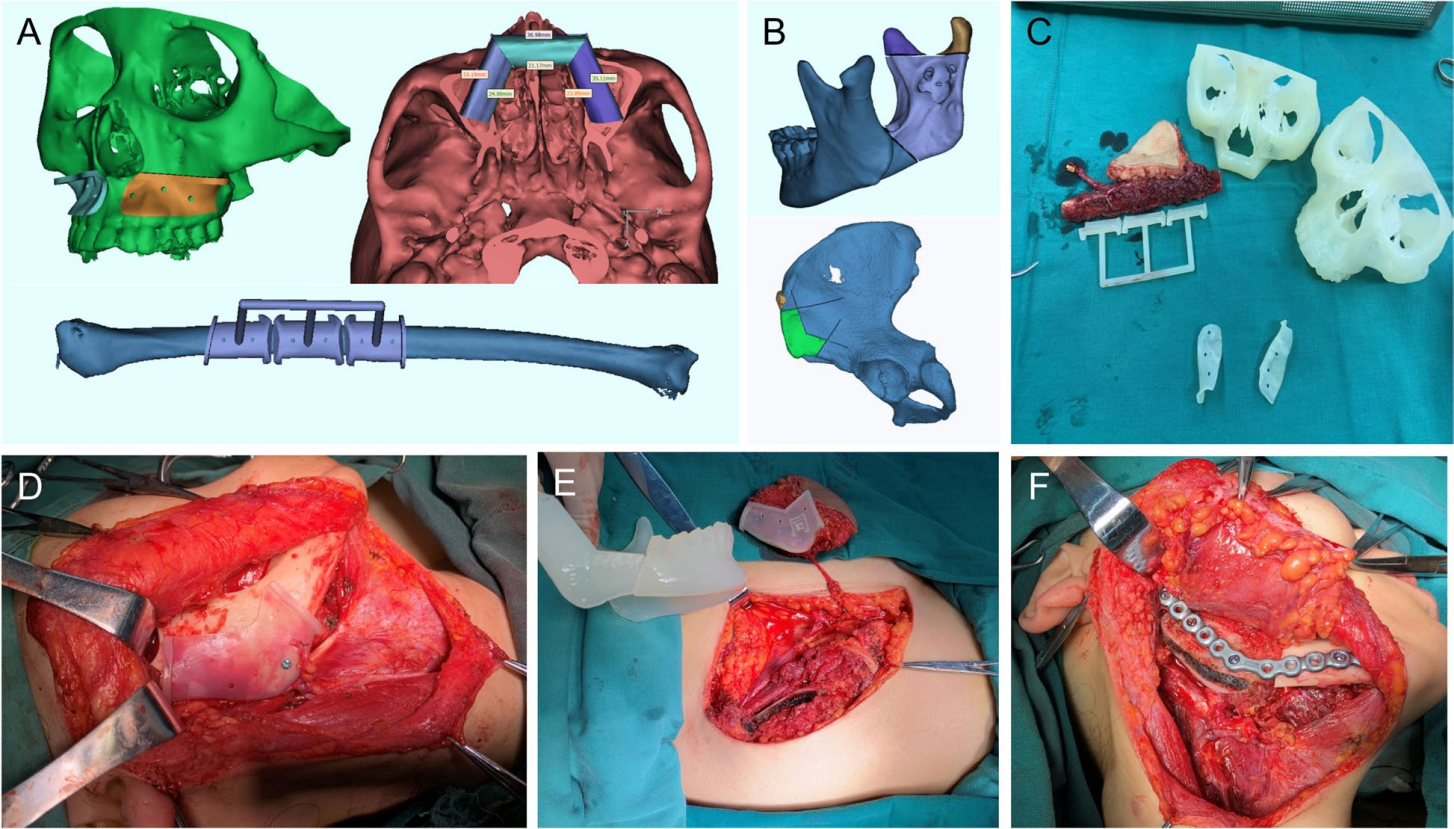
**A** Three-dimensional reconstruction of maxillary lesions by computer simulation. **B** Reconstruction of mandible lesions by computer simulation. **C** 3D printing individual solid model, bone flap solid model, and osteotomy plate. **D** Osteotomy according to the osteotomy guide plate. **E** Design and shaping of the bone flap. **F** The pre-bent titanium plate and pre-determined position of the titanium nail were used for rigid internal fixation

**Fig. 2:**
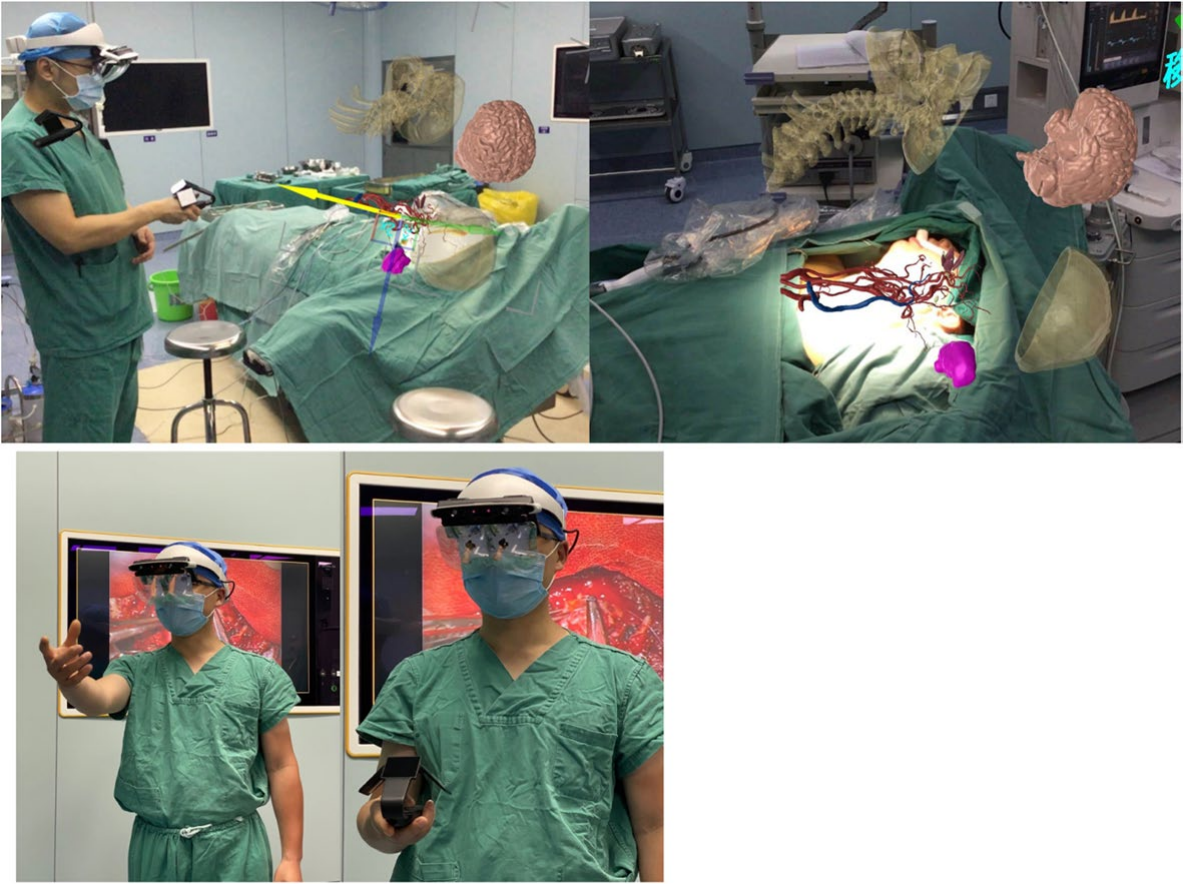
During the operation, the operator can judge the surrounding adjacent and anatomic relationship of tumor, the distance between surgical instruments and tumor, and the degree of tumor resection

Before operation, thin-layer CT scanning was performed on the lesion area to obtain the image data of the lesion location and determine the extent of the lesion tissue. The data in DICOM format is used for linear interpolation to construct a 3D image. After that, median filtering and image enhancement were carried out on the 3D reconstruction image, and the computer 3D visualization reconstruction was carried out (Fig. 1A, B). Then, the data is transmitted to the rapid prototyping system in STL format. The rapid prototyping machine uses its own layering software to relayer and complete the contour editing and molding support setting of the fillings. Then, the CAD/CAM image technology is used to transform the healthy side data into a symmetrical model of the affected side, which is the reference image model after restoration. Finally, the photosensitive resin is used to reconstruct the model. Molecular polylactic acid (PLA) was used as the raw material to produce a three-dimensional individualized solid model, bone flap solid model, and osteotomy plate based on the principle of “layered manufacturing and layer by layer superposition” (Fig. 1C). The resected lesion range was determined according to the above-mentioned three-dimensional reconstruction data and the solid model. The length of the reconstruction titanium plate and the fixed position of the titanium nail were determined according to the virtual operation range and osteotomy plate, the length of the donor site, and the design and molding of the bone flap. Then, the reconstruction titanium plate was pre-bent on the mirror model of the solid model, so as to prepare the bone extraction, shaping, positioning, and fixation according to the template shape during the operation (Fig. 1D–F). According to the simulated occlusal relationship between the upper and lower teeth and the jaw plane, the wax shape was prepared to restore the dentition in the jaw defect area. The working model was turned over and the digital occlusal guide plate was made by pressing film rapid prototyping (Fig. 2).

For patients who need VR, AR, or MR assistance, the DICOM images are further tested to ensure that the images meet quality standards. After special processing of privacy-related data information in these digital pictures, DICOM images were segmented according to different anatomical regions, and the segmented images were reconstructed according to the operation requirements. CT and MRI images were fused by multimodal modeling. The accuracy of the multimodal model is tested by medical imaging experts. The multimodal three-dimensional reconstruction model is transformed into a VR model, and the important structures such as the blood vessel, nerve, bone, and tumor are rendered in different colors to facilitate the identification of parts. The rendered model is imported into a UE4 engine to realize the function customization. By installing cluster rendering equipment, 3D scanner, and 3D scene scanner and wearing a head-mounted display and data glove, surgeons observe the virtual model through holographic VR glasses and use a tablet computer control to match and fuse the virtual model with the patient’s head lesions, so as to quickly locate the body surface projection of the tumor location in the virtual scene using gesture operations to pick up, rotate, scale, split, or profile VR or AR operations (Fig. 2). During the operation, the surgeons can judge the adjacent anatomic relationship of the tumor, the distance between surgical instruments, and the tumor or the degree of resection in real time to implement tumor resection (Fig. 2).

### Evaluating indicator

The location of lesion, pathological type, skin flap selection, complications, osteotomy times, defect range, follow-up time, and survival outcome of the two groups were statistically analyzed (Table 1). At the same time, the short-term and long-term postoperative complications were classified and compared in detail (Table 2). The evaluation was conducted with reference to the EORTC QLQ-H&N Module [9]. In addition, the postoperative functional and aesthetic indexes of the two groups were comprehensively evaluated (Table 3). The subjective evaluation index was mainly conducted by doctors, patients, and family members. The final evaluation result was agreed upon by more than two people, and the EORTC QLQ-H&N Module was also referenced. Objective evaluation indexes included condylar displacement and mandibular angle displacement. Preoperative imaging images were compared with postoperative ones. The displacement exceeding 5mm was considered as deviation. Finally, the time, economic cost, and doctor-patient satisfaction of assisted digital surgical technology were comprehensively evaluated (Table 4). The satisfaction of the doctor-patient relationship was evaluated by the patient-doctor relationship questionnaire-13 (PDRQ-13) [10].

**Table 2:** Postoperative complications and morbidities. Data are given as number of complications

Complications	Digital surgery-assisted (***n*** = 25)	Conventional (***n*** = 27)	***P***-value
**Flap hyperemia or necrosis**	9	8	0.62
**Titanium plate displacement, deformation, or exposure**	3	10	0.03
**Infection**	7	8	0.89
**Orocutaneous fistula**	2	2	0.93
**Weight change**	14	13	0.57
**Hematoma**	6	9	0.46
**Scar deformity**	4	11	0.04
**Constant pain**	6	12	0.12
**Paresthesia**	9	11	0.73
**Cough**	9	6	0.27
**Dry mouth**	12	9	0.28

**Table 3:** Function and aesthetic evaluation of the two group patients. Data are given as number of patients

Variable	Digital surgery-assisted (***n*** = 25)	Conventional (***n*** = 27)	***P***-value
**Speech**			0.09
Ambiguity	2	7	
Understandable	14	16	
Basically normal	9	4	
**Swallow**			0.04
Total oral intake with no restrictions	4	9	
Oral intake with no special preparation, but must avoid specific foods	8	13	
Total oral intake with no restrictions	13	5	
**Eating disorders**	6	15	0.03
**Social barriers**	6	13	0.07
**Opening degree**			0.37
<20 mm	3	7	
20–29 mm	10	11	
≥30 mm	12	9	
**Condylar shift**	4	10	0.09
**Gonion shift**	9	11	0.73
**Malocclusion**	7	10	0.49
**Facial asymmetry**	11	15	0.41

**Table 4:** Time and cost-analysis of digital surgery comparing between two group patients

Variable	Unit	Digital surgery-assisted (mean)	Conventional (mean)	***P***-value
**Total cost of hospitalization**	CNY	88,315	67,599	<0.05
**Operation cost**	CNY	14,784	10,380	<0.05
**Material cost**	CNY	31,010	12,819	<0.05
**Total operation time**	Min	458	532	<0.05
**Molding time of supply area**	Min	34.6	42.5	0.01
**Shaping time of receiving area**	Min	17.1	18.7	0.35
**Cutting or molding time**	Min	12.5	15.0	0.02
**Reconstruction time**	Min	29.4	33.9	0.01
**Vascular anastomosis time**	Min	47.1	49.2	0.50
**Total days in hospital**	Days	19.6	18.1	0.27
**ICU days**	Days	1.36	2.55	<0.05
**Intravenous nutrition support**	Days	7.28	10.1	0.04
**Satisfaction score of doctor-patient relationship**	Score	3.48	2.88	0.02

### Statistical analysis

SPSS 20.0 software was used to test the data. The continuous variables involved in the article were analyzed by an independent sample *t* test. Categorical variables were tested by a chi-square test. *P* < 0.05 was considered statistically significant.

## Results

### Comparison of main clinicopathological features

A total of 52 patients were enrolled in this study, including 25 patients in the DS group and 27 patients in the CS group. The average age of the DS group (45.7 years old) was slightly lower than that of the CS group (48.8 years old), but this difference was not statistically significant. In addition, there were no significant differences in gender, smoking status, pathological type, lesion distribution, complications, or adjuvant treatment between the two groups. There were no significant differences in the factors related to the operation, including the type of flap, the size of the lesion after resection, neck lymph node dissection, osteotomy times, and reoperation exploration rate. However, in the mean value of the lesion range after resection, the DS group (6.2cm) was larger than the CS group (5.7cm). In addition, there was no significant difference in survival outcome and follow-up time between the two groups. The results showed that the two groups of patients did not interfere with the main evaluation indicators of this study, and the two groups were comparable, as shown in Table 1.

### Comparison of complications

By comparison, only 3 patients in the DS group supplemented with digital surgical techniques experienced titanium plate displacement, deformation, or exposure, and the incidence was significantly lower than that in the conventional empirical repair group (*P* < 0.05). In addition, the incidence of facial scar deformity in the DS group was significantly lower than that in the CS group (*P* < 0.05). However, in other short-term or long-term postoperative complications (such as flap congestion or necrosis, infection, orocutaneous fistula, hematoma, weight change, persistent pain, paresthesia, cough, and dry mouth), although some indicators in the DS group were slightly lower than those in the CS group, there was no statistically significant difference (*P* > 0.05), as shown in Table 2.

### Functional and aesthetic evaluation

Just by comparison, the incidence of swallowing and eating disorders in patients assisted by digital surgical technology was significantly reduced (*P* < 0.05). There was some improvement in postoperative speech and social function, but the statistical results were not significantly different (*P* > 0.05). In terms of facial aesthetic evaluation, there was no significant difference between the two groups in opening degree, condylar shift, gonion shift, malocclusion, and facial asymmetry (*P* > 0.05). Details are shown in Table 3.

### Time-economic-social benefit evaluation

The results of this study show that patients with digital surgical assist technology have higher total hospitalization costs, surgical costs, and material costs than the traditional empirical repair group (*P* < 0.05). However, the total operation time, the preparation time of bone flaps in the donor area, osteotomy time, and reconstruction time were significantly lower than the traditional surgery group (*P* < 0.05), and it could not shorten the shaping time of the receiving area and the time of vascular anastomosis (*P* > 0.05). In addition, the total hospitalization days of the two groups were not obvious differences, but the time of ICU treatment and the time of postoperative intravenous nutritional support of patients in the DS group are shorter than those of the traditional surgery group (*P* < 0.05). It is particularly worth mentioning that in the process of digital surgical assisted treatment, due to the presentation of three-dimensional images and 3D printed physical models, and even the intervention of immersive VR technology, the preoperative doctor-patient communication of this group of patients is more effective, and the treatment satisfaction of patients and their families after surgery is higher (*P* < 0.05).

### Typical cases of comprehensive application of 3D reconstruction, digital simulation, and 3D printing technology

**Fig. 3:**
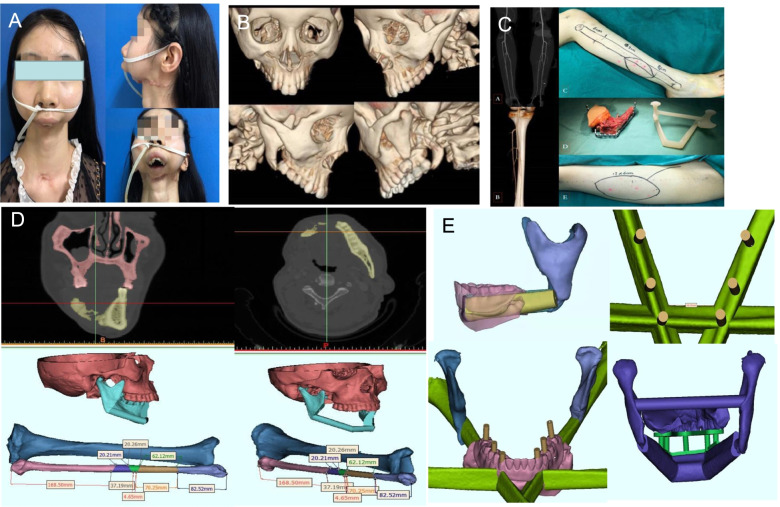
**A** Preoperative facial features of patients. **B** Three-dimensional reconstruction of maxillofacial CT before operation. **C** The three-dimensional solid model and osteotomy plate of the vascular perforator, lesion site, and fibula. **D** Computer-aided design to simulate the osteotomy range of fibula flap and mandible reconstruction. **E** Based on the three-dimensional reconstruction model image, the implant restoration guide plate was designed and 3D printed

**Fig. 4:**
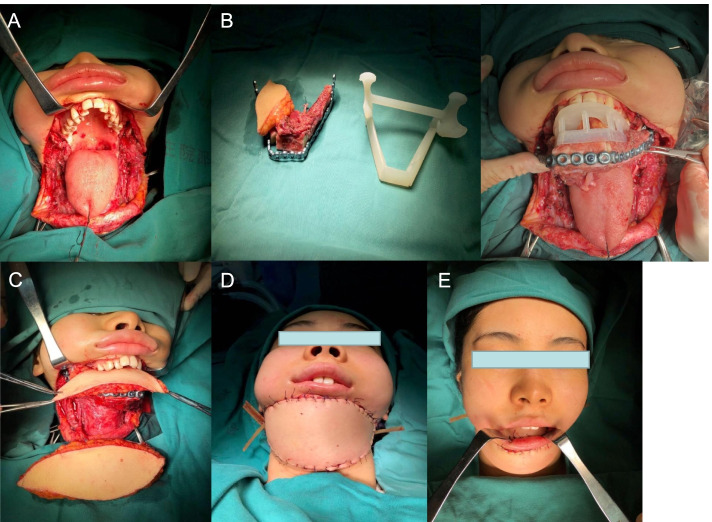
**A** Mandibular defects and soft tissue defects were found during the operation. **B** The relationship between free fibula and maxilla and the position of titanium screws were determined by implant repair guide plate, and then the titanium plate with suitable length and radian was molded and fixed in the predetermined position by using preoperative computer simulation and 3D physical model. **C** The peroneal flap was used to reconstruct the floor of the mouth, and the lateral femoral flap was used to reconstruct the neck defect. **D** Status after intraoperative reconstruction. **E** Intraoral conditions after intraoperative reconstruction

**Fig. 5:**
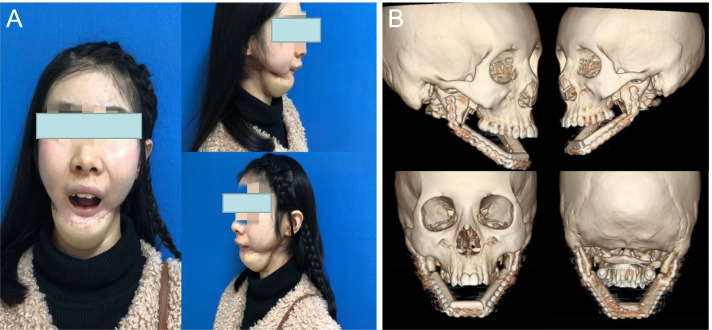
**A** The gastric tube was removed 3 months after the operation, and the opening was about 5 cm. **B** Three-dimensional reconstruction of CT scan 3 months after the operation

The patient, a 21-year-old female, was diagnosed with bilateral mandibular osteosarcoma 5 years ago. The patient required radiotherapy with a total dose of 104 Gy in twice. Three years ago, the tumor reoccurred and required surgical treatment, and the free rib necrosis resulted in complete mandibular defect (Fig. 3A). Two years ago, she was treated in our hospital for secondary mandibular reconstruction. The treatment plan was discussed by the multidiscipline team (MDT) several times before the operation. The maxillofacial and bone three-dimensional reconstruction was performed to evaluate the extent of jaw defect (Fig. 3B). Meanwhile, the perforators of fibula and anterolateral femoral vessels in the donor site were identified (Fig. 3C). CAD technology combined with 3D printing technology was used to assist in the formulation of the surgical plan. According to the mandible defect area and fibula width, thickness and angulation data were compared to determine the optimal fibula osteotomy area and to do virtual reconstruction (Fig. 3D). Each cross-section was formed by a 3D printer, and the 3D solid model and osteotomy plate were gradually superimposed in proportion to the lesion site and fibula (Fig. 3C). According to the reconstructed model of rapid prototyping, titanium plates with suitable length and angle were prepared before operation. At the same time, the 3D reconstruction model image was used to design and 3D print the implant restoration guide plate (Fig. 3E). According to the preoperative simulated operation plan, the right fibular myocutaneous flap free reconstruction of mandible + left lateral femoral transplantation + vascular anastomosis + titanium plate internal fixation + tracheotomy was performed under general anesthesia. The relationship between free fibula and maxilla and the position between titanium nail and screws were determined by an implant reconstruction guide plate. After the occlusal relationship was determined, the titanium plate with suitable length and radian was molded and fixed in the predetermined position which was confirmed by computer simulation and 3D physical model before the operation (Fig. 4). The patient recovered well afterwards. A small amount of oral liquid diet started 1 month after the operation, and the oral liquid diet was completely consumed after 3 months (Fig. 5A). The examination showed that the reconstructed mandible grew well (Fig. 5B). At present, the patient is currently undergoing later functional exercise, and the opening degree is about 5cm. The dental implant restoration will be completed in the later.

### Typical cases using VR, MR, and other techniques to assist surgical treatment

**Fig. 6:**
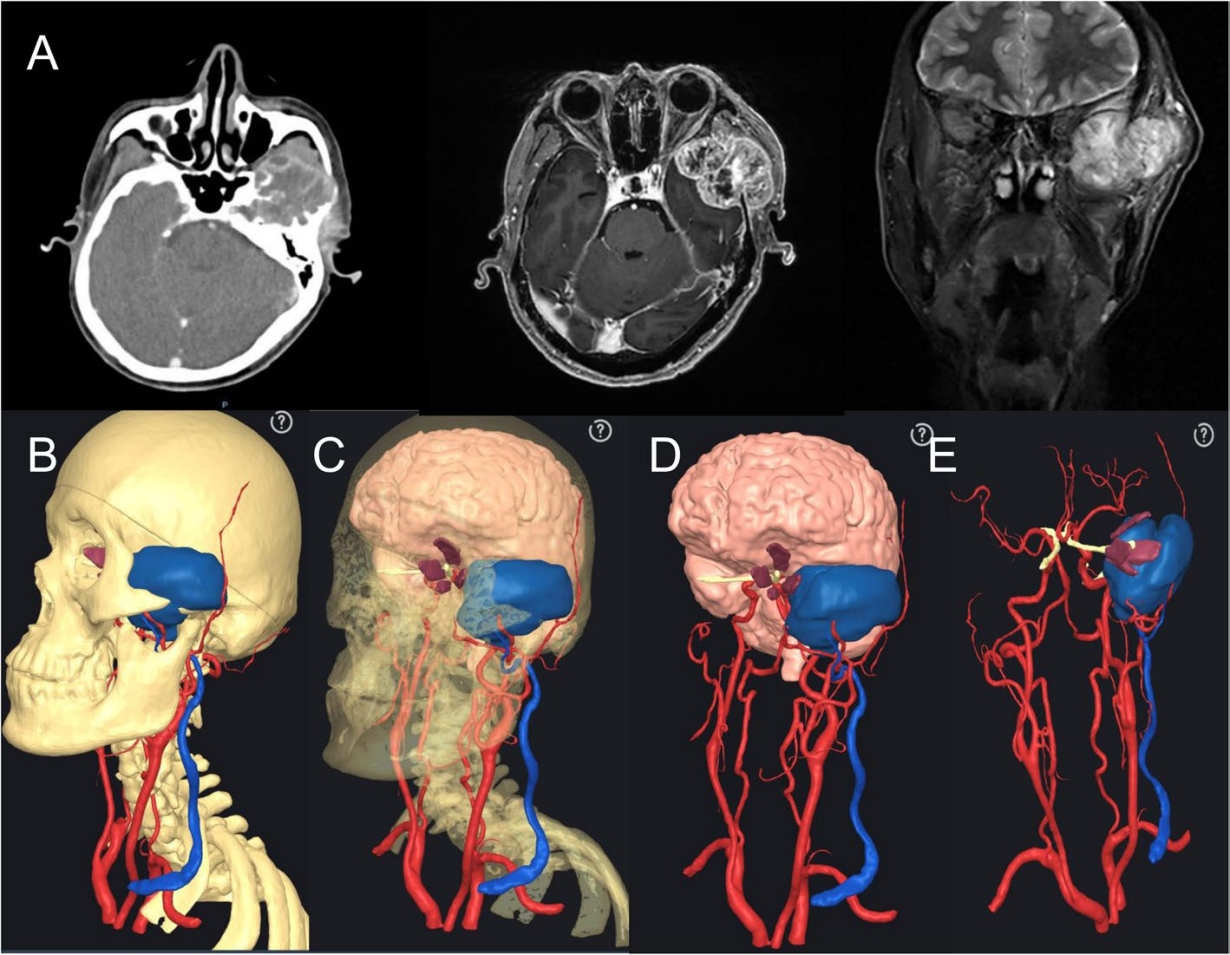
**A** Preoperative enhanced CT and MRI imaging showed the extent of the lesion. **B** Three-dimensional reconstruction after skin and soft tissue removal. **C** The relationship between the tumor and adjacent tissues was demonstrated by crypto jaw enucleation and skull transparency. **D** Saphenous osteotomy showed tumor and important structures (only blood vessels, brain, and nerves). **E** The tumors and important structures (only blood vessels and nerves) can be shown in the brain tissue

**Fig. 7:**
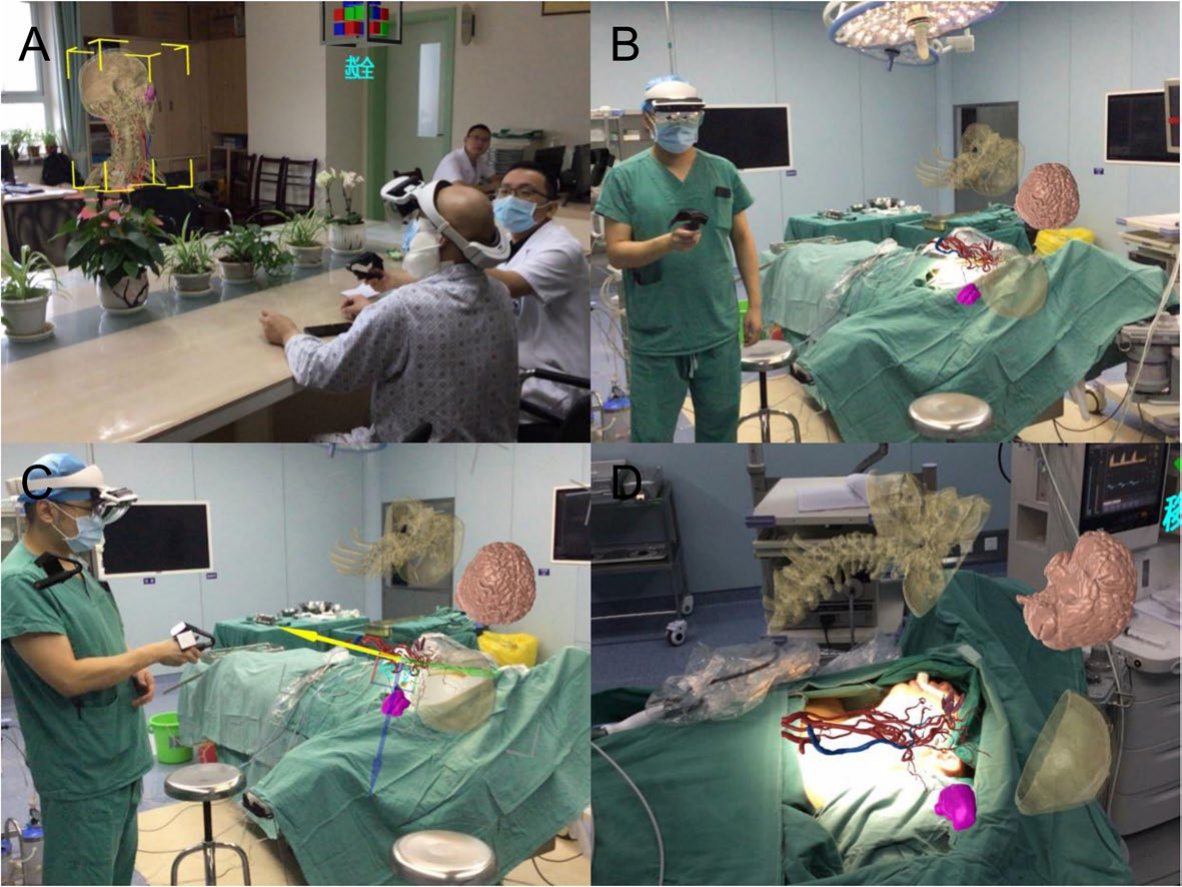
**A** Preoperative use of MR technology and patients with accurate, effective visual communication between doctors and patients. **B**-**D** In the course of the operation, the MR technique was used to locate, split, and monitor the tumor in real time

**Fig. 8:**
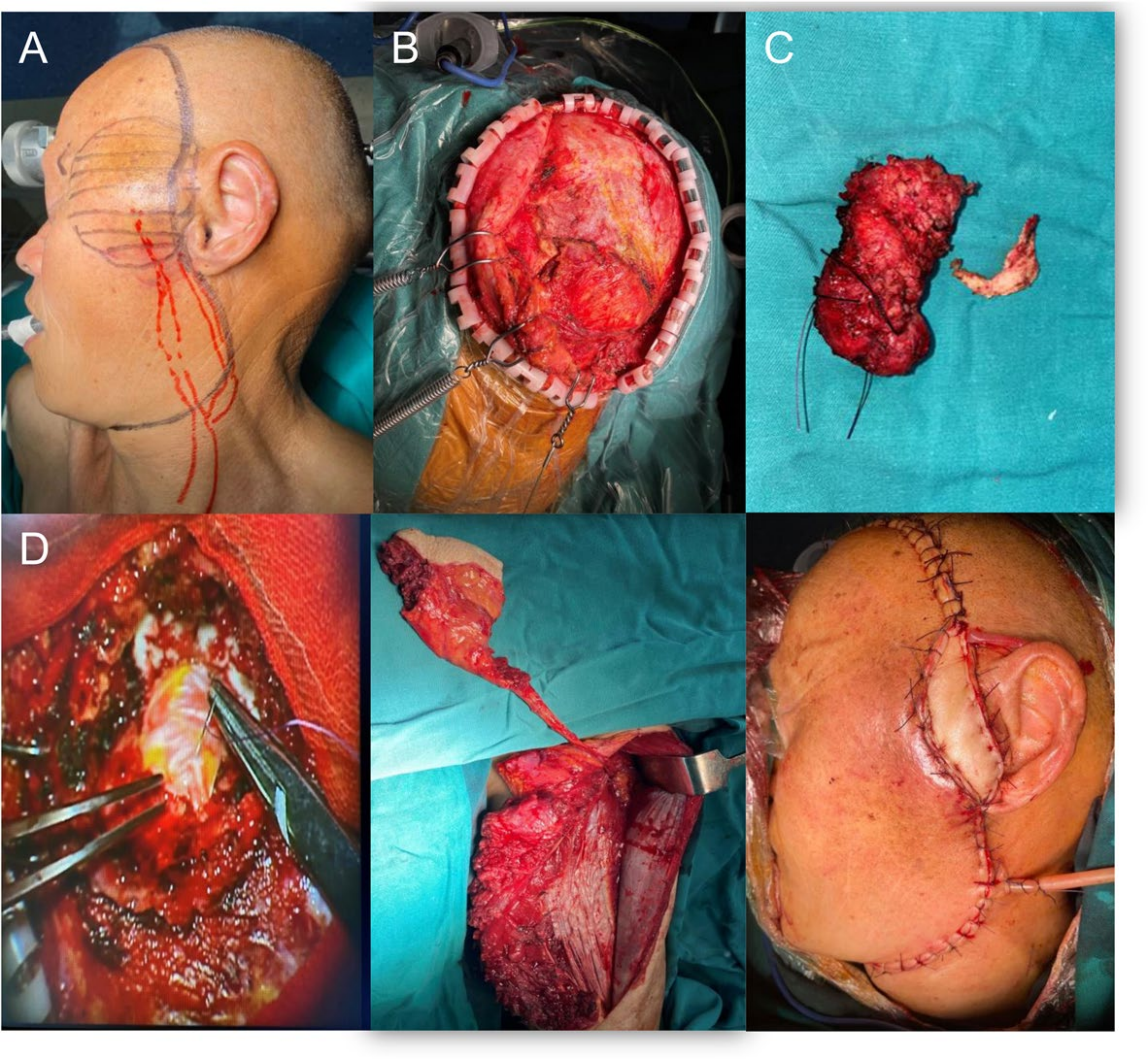
**A** Preoperative tumor location and surgical procedure design. **B** The exposure of primary lesion in operation. **C** According to the operation plan, the tumor and zygomatic arch were completely removed under the guidance and assistance of MR. **D** The lateral femoral myocutaneous flap was used to repair the defect in the operation area (the muscle flap was used to fill the lateral skull base, the fascia lata was used to repair the dura mater, and the skin island was used as the observation window of the myocutaneous flap)

A 69-year-old male patient presented with a progressive mass in the left temporal region. The cytological and pathological diagnosis obtained by coarse needle aspiration showed malignant solitary fibrous tumor in the left temporal region. Enhanced CT and MRI showed that the left temporal intracranial and infratemporal fossa areas found irregular soft tissue masses. Bone was destructed in the zygomatic process, squama of the left temporal bone, and the greater wing of the sphenoid bone. The lesions may invade the left lateral pterygoid muscle and temporal muscle, and the left temporal lobe is compressed (Fig. 6A). The three-dimensional reconstruction showed that the tumor had abundant blood supply, with branches of the external carotid artery, especially in the periphery, pushing the adjacent left external jugular vein and its branches (Fig. 6B–D). Before operation, the patient was considered to have a complex craniomaxillofacial communication tumor. VR technology was used to communicate with patients accurately, vividly, and effectively, so that patients could understand their own condition, operation purpose, and risk (Fig. 7A). After completing the preoperative examination, “left lateral skull base communication tumor resection + left neck lymph node dissection + right lateral femoral free muscle flap repair + temporal bone and sphenoid greater wing partial resection + zygomatic partial resection + dural partial resection + right leg broad ligament dural repair + small blood vessel anastomosis” were performed under general anesthesia (Fig. 8). During the operation, MR technology was used to locate, split, and monitor the tumor in real time which can assist the operation safely (Fig. 7B, C). The patient was discharged from the hospital 9 days after the operation followed with adjuvant treatment, such as radiotherapy and chemotherapy, and recovered well.

## Discussion

For a long time, the treatment of head and neck tumors has been based on surgery, supplemented by radiotherapy and chemotherapy. In recent years, with the rapid development of immunotherapy and targeted therapy, the treatment of head and neck tumors has entered a new era. The comprehensive application of various means has improved the survival rate of patients and preserved function greatly. However, due to the low level of immune expression or the mutation of important gene loci in some patients, the therapeutic effect of new methods is seriously affected [11, 12]. Surgery, as the earliest and most important treatment method, is still the most important factor affecting the therapeutic effect. It has always been the goal of head and neck surgeons to maximize tumor resection and obtain a reliable and safe boundary. However, due to its special location and anatomy, it often needs reconstruction when the tumor is removed from the head and neck. Most small defects can be repaired with adjacent flaps or pedicled flaps [13]. Medium and large defects are mainly repaired with the pedicled flap and free flap. Although some scholars have made in-depth research and improvement on the pedicled flap in the past to make its indication wider, safer, and more reliable [14, 15], the free flap has gradually become the main choice for repair and reconstruction of these patients because of its unique advantages, especially when it involves bone tissue reconstruction. However, for the free flap, the efficiency and accuracy of traditional reconstruction are not ideal, which restricts its clinical application. With the development and application of various digital surgical techniques, the reconstruction after head and neck tumor resection has embarked on a precise and individualized road.

Three-dimensional reconstruction technology is the cornerstone of digital surgical technology, and it has a milestone significance as the beginning of digital technology. Three-dimensional reconstruction technology can not only provide intuitive, clear, and specific three-dimensional tissue images, but also can be controlled by doctors through digital software to make them move. Thus, doctors can observe the lesions and their adjacent relationship with the surrounding important anatomical structures from various angles, which are more intuitive. Surgeons can also judge the location and range of tumors so as to determine the safe boundary for tumor resection. It is obvious that the technology provides strong support for accurate diagnosis and treatment [16].

Three-dimensional reconstruction technology is also widely used in clinical practice. It is also one of the earliest digital technologies applied in our department. At present, patients with advanced head and neck malignant tumors who need surgical treatment in our department are treated with three-dimensional reconstruction routinely when important adjacent tissues or structures of the head and neck are involved. In this study, all patients in the DS group used 3D reconstruction technology before the operation. With this technology, we can accurately reconstruct the three-dimensional structure of the head and neck with computer software and judge the location, range, invasion, and destruction of bone tissue in the 3D reconstruction model. Intuitive three-dimensional images help the doctor to design the operation and implement accurate resection (Fig. 3B). At the same time, it is helpful for surgeons to explain the operation plan to patients before the operation to promote communication between doctors and patients. Our results also show that the combination of 3D reconstructed images and 3D printed models can effectively improve the postoperative satisfaction of patients.

CAD technology is based on imaging data, which in the digital software for virtual design of surgical process. In our clinical work, we found that in the traditional treatment mode, the resection range of head and neck tumor is usually roughly determined by the operator according to clinical examination and imaging examination, which lacks personalized surgical design. With the prevalence of vascularized free tissue flap repair and reconstruction, for example, when the tumor area is large and the maxilla and mandible are involved, the appearance of the patients is obviously affected. The restoration of the occlusion relationship and the three-dimensional position of the grafts are usually determined by the experience of the operator. However, this “experience dependent” positioning method usually lacks sufficient accuracy and stability, resulting in inaccurate head and neck reconstruction and transplantation. If the three-dimensional position of bone reconstruction is not ideal, it is difficult to obtain a satisfactory occlusal relationship and masticatory function recovery, and cannot achieve the precise reconstruction of personalized function desired appearance.

In addition, CAD technology enables doctors to use various digital software to carry out tumor resection and reconstruction in the virtual model before the operation. According to the nature and three-dimensional position of the tumor, the scope of resection was determined, and especially when bone reconstruction is involved, the position of the osteotomy line is designed in the digital software for virtual osteotomy [17]. In particular, the mandible has a unique horseshoe-shaped structure. Mandibular tumors usually lead to destruction of the mandible bone, so it is difficult to restore the normal shape of the mandible [18]. In the cases of mandibular reconstruction we provided, we used CAD technology to simulate the whole mandible before the operation, and restored the pre-amputation shape of the mandible according to the data stored in the digital software. According to the range and location of the defect, the length and angle of each segment of bone graft were precisely designed to meet the needs of shape and function repair in a three-dimensional position (Fig. 1A, B).

The core of CAM technology is 3D printing technology. In recent years, there are more and more auxiliary applications in head and neck surgery. This technique is one of the important ways to transform the preoperative design into the actual operation process. At the same time, we can design the osteotomy guide plate, shaping guide plate, and other surgical guide plates simultaneously while making the virtual design scheme for patients with head and neck tumor involving the jaw bone and manufacture the same proportion of real objects through 3D printing technology. In the process of mandible osteotomy, fibula cutting, and shaping, the corresponding 3D printing guide plate is used to guide the operation, so as to transform the preoperative virtual design scheme into the actual operation. Through this method, we can achieve the goal of precise resection and accurate reconstruction. In addition, according to the preoperative design, the reconstruction model of the mandible after fibula or iliac reconstruction can be printed out, and personalized reconstruction titanium plate can be pre-bent to guide the accurate restoration of jaw shape and occlusion relationship (Figs. 1C and 3C), which reduces the dependence on subjective experience, while simplifying and reducing the difficulty of the operation. Some studies [19] compared the coincidence degree between the preoperative design and postoperative actual mandible, fibula osteotomy line, and reconstruction titanium plate. It was considered that the maximum error of reconstruction came from the manual bending reconstruction titanium plate, which also proved the reliability of digital surgical technology applied to jaw defect reconstruction. CAD/CAM technology is used in most cases in this study. The results of this study also show that the pre-bent titanium plate can better shape and reduce the stress of the titanium plate. It also reduces the risk of titanium plate displacement, deformation, and exposure after surgery. Patients could obtain better facial appearance and reduce unnecessary broad scar. Other surgical-related complications also decreased from the study results, although there was no statistically significant difference.

It must be pointed out that although CAD/CAM technology has many advantages, it still has some shortcomings and limitations in our clinical work. The existing CAD technology is mainly based on bone tissue structure, which cannot accurately estimate and judge the influence of soft tissue. Many types of surgical guides are designed based on bone structure. In the actual operation process, the influence of soft tissue cannot be ignored. On the one hand, the existence of soft tissue affects the preoperative judgment of the tumor range. Once it is found that the actual resection range needs to be changed during the operation, the surgical guide plate will not be used; on the other hand, it will affect the fitting degree of the surgical guide plate and bone tissue, which will affect the accuracy of the operation to a certain extent.

In order to accurately estimate and judge the influence of soft tissue and solve the above problems, VR, AR, and MR techniques have been gradually used in head and neck surgery in recent years. These technologies can provide “perspective function,” which is equivalent to wearing a “perspective eye” for doctors. Earlier, our department took the lead in the application of VR technology in preoperative evaluation and simulation and achieved some results [20]. Recently, we increased the application of MR technology in the reconstruction surgery, adjusted the imaging model of the patient, used the navigation technology embedded matching positioning on the patient, and operated the operation through the panoramic stereo imaging in the AR glasses. Moreover, with the displacement of the patient’s position during the operation, the image can still maintain accurate positioning to assist the implementation of the precise operation (Fig. 7B, C). After our exploration, we found that the main advantages of this kind of technology are (1) the new imaging technology can form embedded three-dimensional images, which can display the complex soft tissue situation in front of clinicians more intuitively; (2) do not need to rely on the doctor’s subjective impression to avoid the surgical risk caused by visual gaps; and (3) reflect the osteotomy angle and osteotomy line planned before surgery in the operation.

In addition to the above-mentioned digital surgical techniques commonly used in our department, with the development of science, technology, and economy, more and more emerging technologies are used for precise repair and personalized reconstruction of head and neck tumors. Surgical navigation technology is another effective way to transform a virtual design into surgical practice. Compared with the surgical guide plate, the advantage of the navigation technology is that it can carry out “real-time” verification and guidance in a three-dimensional position. The preoperative design of the surgical scheme can not only be carried out accurately, but also be flexibly adjusted according to the actual situation [21]. Three-dimensional measurement and evaluation technology can match the reconstructed jaw model with the ideal designed model before operation and analyze the reconstruction error by chromatographic analysis. The difference of the coordinates and angles of the main landmarks before and after the operation was compared to evaluate the accuracy of the repair and reconstruction. This method provides a quantitative evaluation standard for the reconstruction effect and can intuitively help the surgeon to find intraoperative problems in order to better advance and improvement [22]. The surgical robot has a “sensing system” and a “vision system,” which can accurately locate the operation position according to the preoperative design. In recent years, it has been used in a variety of head and neck surgery and achieved a good therapeutic effect [23]. However, there are few reports on the reconstruction of jaw defects. In recent years, it has been reported that the model and animal experiment of robot-assisted free fibula flap for mandibular defect repair [24]. The experimental results show that the repair accuracy of the surgical robot is significantly better than that of traditional surgery, which confirms the application value and advantages of surgical robots in mandibular reconstruction surgery.

The application of these digital surgical technologies can not only ensure the safety of surgery, reduce the difficulty of surgery, improve the effect of repair, and facilitate the postoperative function, but also shorten the process of surgery and the recovery time of patients to a certain extent. However, it must be pointed out that our research results show that due to the high equipment cost, material cost, and labor cost of these technologies, although they have many advantages and can save the time cost of doctors and patients, this approach also significantly increases the cost of surgery and hospitalization for patients. Therefore, in the process of clinical application, we can not only consider the advantages of the technology itself, but also combine the patient’s own situation and economic conditions to selectively use effective digital technology. After all, compared with conventional experience repair, most of the indicators in this study, especially in the repair effect and the incidence of complications, have shown certain advantages, but there is no significant statistical difference. Of course, the limited sample size and retrospective research methods limit the stability and reliability of the research results. At the same time, the development of digital technology has a process, so there may be differences in the time sequence and social status of patients. In order to obtain more specific, accurate, and comprehensive evaluation results, we need to further carry out large sample randomized controlled trials to verify. It is safe to say that under the guidance of the concept of precision medicine, with the development of digital technology and the efforts of clinicians, the level of head and neck reconstruction is bound to be raised to a new height.

## Conclusion

Comprehensive application of digital surgical technology in the reconstruction of the head and neck after tumor resection is feasible in clinical practice, which can not only improve the accuracy of repair, decrease some surgical complications, better preserve and improve the patient’s diet and speech function, and reduce the operation and hospitalization time, but also increase the treatment cost. Furthermore, it is conducive to doctor-patient communication and improves patient satisfaction. Digital surgical technology can be used as an auxiliary method for head and neck reconstruction.

## Data Availability

The datasets used or analyzed during the current study are available from the corresponding authors on reasonable request.

## Abbreviations

DS: Digital surgery
CS: Conventional surgery
CAD: Computer-aided design
CAM: Computer-aided manufacturing
VR: Virtual reality
AR: Augmented reality
MR: Mixed reality
